# Expression of CCL20 and Its Corresponding Receptor CCR6 Is Enhanced in Active Inflammatory Bowel Disease, and TLR3 Mediates CCL20 Expression in Colonic Epithelial Cells

**DOI:** 10.1371/journal.pone.0141710

**Published:** 2015-11-04

**Authors:** Helene Kolstad Skovdahl, Atle van Beelen Granlund, Ann Elisabet Østvik, Torunn Bruland, Ingunn Bakke, Sverre Helge Torp, Jan Kristian Damås, Arne Kristian Sandvik

**Affiliations:** 1 Centre of Molecular Inflammation Research, Norwegian University of Science and Technology, Trondheim, Norway; 2 Department of Cancer Research and Molecular Medicine, Norwegian University of Science and Technology, Trondheim, Norway; 3 Department of Laboratory Medicine, Children and Women’s Health, Norwegian University of Science and Technology, Trondheim, Norway; 4 Department of Gastroenterology and Hepatology, St. Olav’s University Hospital, Trondheim, Norway; 5 Department of Pathology and Medical Genetics, St. Olav’s University Hospital, Trondheim, Norway; 6 Department of Infectious Diseases, St. Olav’s University Hospital, Trondheim, Norway; University of South Carolina School of Medicine, UNITED STATES

## Abstract

**Background:**

The chemokine CCL20 and its receptor CCR6 are putative drug targets in inflammatory bowel disease, and CCL20 is a novel IBD predilection gene. Previous findings on the CCL20 response in these diseases are divergent. This study was undertaken to examine CCL20 and CCR6 during active and inactive disease, and mechanisms for CCL20 regulation by the innate immune system. As TLR3 has recently emerged as a possible mediator of CCL20 production, we hypothesised that this TLR plays an important role in enterocytic CCL20 production.

**Methods:**

A large microarray study on colonic pinch biopsies from active and inactive ulcerative colitis and Crohn’s disease provided background information. CCL20 and CCR6 were localized and their expression levels assessed in biopsies using *in situ* hybridization and immunohistochemistry. Regulation of CCL20 was studied in the HT29 cell line using a panel of pattern recognition receptor ligands followed by a TLR3 siRNA assay.

**Results:**

*CCL20* and *CCR6* mRNA abundances were increased during active inflammation (*CCL20* 5.4-fold in ulcerative colitis and 4.2-fold in Crohn’s disease; *CCR6* 1.8 and 2.0, respectively). *CCL20* and *CCR6* mRNA positive immune cells in lamina propria were more numerous, and CCL20 immunoreactivity increased massively in the epithelial cells during active inflammation for both diseases. TLR3 stimulation potently induced upregulation and release of CCL20 from HT29 cells, and *TLR3* silencing reduced CCL20 mRNA and protein levels.

**Conclusions:**

The CCL20-CCR6 axis is involved during active inflammation in both ulcerative colitis and Crohn’s disease. The epithelial cells seem particularly involved in the CCL20 response, and results from this study strongly suggest that the innate immune system is important for activation of the epithelium, especially through TLR3.

## Introduction

Ulcerative colitis and Crohn’s disease, collectively termed inflammatory bowel disease (IBD) affect approximately 2.2 million Europeans. Although these diseases are a huge burden for individuals and for the society [1], their aetiology and pathogenesis are still disputed. The current understanding of IBD is that the disease is a response to environmental factors that are normally tolerated, such as commensal microbes, in genetically predisposed individuals. Much of the observed genetic predisposition concerns genes related to the innate and adaptive immune systems [2], and an important element in the efforts to understand the disease pathogenesis is to characterize the extremely complex immune and inflammatory responses in the diseased gut [3]. The pattern recognition receptors (PRR) play an integral part in these responses. These are receptors of the innate immune system, which recognize specific components of foreign material; pathogen-associated molecular patterns (PAMPs) and danger-associated molecular patterns (DAMPs). This pattern recognition results in an appropriate regulation of the inflammatory response [4]. The toll-like receptors (TLR) were the first PRRs to be discovered, and in humans there are 10 functional TLRs [5]. The role of PRRs in regulating inflammation through modulating cytokine production, and their contribution to the pathogenesis of IBD has been widely studied [6].

The chemokine CCL20 is of particular interest in IBD, due to its role in shaping gut immunity [7, 8]. Moreover, CCL20 was recently identified as a susceptibility gene for IBD, adding to the interest in its role in these diseases [9]. Targeting CCL20 and CCR6 has been suggested as new treatment strategies in autoimmune diseases [10–12], and their role in IBD needs to be clarified. CCL20 is a C-C-L chemokine containing the four characteristic cysteine residues with a well-conserved relative distance, and binds exclusively to the CCR6 receptor [13]. CCL20 is constitutively expressed by neutrophils, enterocytes, B-cells and dendritic cells, and by even more cell types when stimulated with proinflammatory ligands, while CCR6 is expressed by T regulatory (Treg), T helper type 17 (Th17) and immature dendritic cells, as well as B-cells [14]. CCL20 has been shown to direct Treg, Th17, B-cells and immature dendritic cells to the gut mucosa [14–19]. There are two known aspects of CCL20 action; one is the chemokine function as described, the second an antimicrobial effect related to the beta defensins, which are also ligands of CCR6 [20–22]. The possible important role of TLRs in CCL20 release was shown when Sugiura et al. found that TLR1 stimulation mediated CCL20 release [23]. TLR3 has recently emerged as an interesting factor in IBD pathogenesis, and the production of CCL20 from gingival fibroblasts after TLR3 stimulation was the first tie between CCL20 and TLR3 [24].

A genome-wide gene expression study on IBD colonic mucosa from our laboratory combined with a meta-analysis of comparable datasets pointed out the chemokine CCL20 and its receptor CCR6 as potentially important in the pathogenesis of these diseases [3]. The aim of the present study was to further explore the expression of CCL20 and CCR6 in IBD by localizing these in the colonic mucosa, and also to investigate the mechanisms for CCL20 regulation. In particular, we have investigated how a broad panel of PRR ligands regulate CCL20 synthesis and release in colonic epithelial cells, with the hypothesis that TLR3 plays an important role in enterocytic CCL20 production.

## Materials and Methods

### 2.1 Ethical considerations

All subjects included in the study gave informed written consent. The study was approved by the Regional Medical Research Ethics Committee of Central Norway (Ref. no. 5.2007.910), and registered in the Clinical Trials Protocol Registration System (identifier NCT00516776).

### 2.2 Patients

Patients undergoing colonoscopy for known or suspected IBD at the Gastrointestinal Endoscopy Unit, St. Olav’s University Hospital, Trondheim, Norway, were included in the study. Healthy controls were recruited among persons undergoing colonoscopy due to gastrointestinal symptoms with no gastrointestinal disease found (Table 1).

**Table 1:** Characteristics of subjects enrolled in microarray analysis.

	Controls	UC	CD	P
Number of subjects	20	49	23	
Age, years(range)	45(19–71)	45(19–72)	36,7(20–63)	n.s
Female sex (%)	9(45)	24(49)	9(39)	n.s
Duration of disease, years (range)	-	14(0,25–40)	7(0,2–28)	n.s
5-ASA/S-ASA (%)	0	34(69)	7(30)	0.002*
Systemic corticosteroids (%)	0	9(18)	9(39)	n.s

Age and duration of disease are given as mean and gender and medication as numbers. UC = Ulcerative colitis. CD = Crohn's disease.

* Significantly higher use of 5-ASA/S-ASA in UC vs CD subjects.

Pinch biopsies were collected from endoscopically assessed maximally inflamed colonic mucosa, and normal biopsies from the hepatic flexure for all patients (n = 92). Four adjacent biopsies were taken from each site, and either formalin-fixed or snap-frozen and stored in liquid nitrogen. Diagnosis was confirmed by assessment of hematoxylin-eosin stained sections by an expert pathologist, and samples where endoscopic and microscopic assessment differed were excluded from the study. The subjects were divided into five groups: controls (N), active ulcerative colitis (UCa), inactive ulcerative colitis (UCi), active Crohn’s disease (CDa) and inactive Crohn’s disease (CDi). The clinical material is further described by Granlund et al [3].

### 2.3 Procedure

#### 2.3.1 Transcriptome analysis and qRT-PCR confirmation

Total RNA was isolated from snap frozen biopsies using Ambion mirVana total RNA Isolation Kit (Applied Biosystems, CA, USA). Illumina Human HT-12 expression Bead-Chips (Illumina, San Diego, CA, USA) was used for global transcriptome analysis. This has been described in detail previously[3] and the full data set is available at ArrayExpress E-MTAB-184.

Confirmative qRT-PCR was done on cDNA reverse transcribed using the High Capacity RNA-to-cDNA kit (Applied Biosystems, Foster City, CA, USA) according to the manufacturer’s recommendations. For *CCL20*, a TaqMan assay was used with the Fast Real-Time PCR Universal PCR Master Mix and TaqMan probes (probe ID Hs01011368_m1, Life Technologies, CA, USA). *CCR6* qRT-PCR analysis was done with the PerfeCta SYBR Green FastMix, ROX (Quanta Biosciences, Gaithersburg, MD, USA), forward primer CCTAGCGGAGTTCCAGCAAA and reverse primer: AATTCCAGCTGTCCCCTAGC (Life Technologies, Paisley, UK). All PCR procedures were done using the StepOnePlus Real-Time PCR System and StepOne software v. 2.1, with glyceraldehyde 3-phosphate dehydrogenase (*GAPDH*) as housekeeping gene (Taqman probe ID Hs99999905_m1, SYBR green primers forward: GCCGCATCTTCTTTTGCGTC reverse: GATCTCGCTCCTGGAAGATGG, Life Technologies, Paisley, UK).

#### 2.3.2 Histological examination, immunostaining and *in situ* hybridization

Four micron sections were made from formalin-fixed colonic biopsies embedded in paraffin. Hematoxylin and eosin stained sections were classified as”normal”,”chronic inflammation” or”chronic active inflammation” based on infiltration of neutrophils and mononuclear cells.

Immunohistochemistry (IHC) for CCL20 and CCR6 was done on randomly selected biopsies, using samples from five subjects from each of the groups UCa, UCi, CDa, CDi and N. Primary antibodies for both CCL20 (rabbit polyclonal antibody, cat. no. Ab9829, Abcam, Cambridge, UK) and CCR6 (rabbit polyclonal antibody, cat.no. PA5-29015, ThermoFisher Scientific, Pierce Biotechnology, Rockford, IL, USA) were used 1:400 in PBS with 1% bovine serum albumin (BSA). Mouse antibodies (all from Dako, Glostrup, Denmark) used for determination of cells types in lamina propria were CD3 (T-cells, 1:100, M7254), CD20cy (B-cells, 1:300, M0755), CD45RO (activated T-cells, 1:300, M0742), and CD1α (immature dendritic cells, 1:300, M3571) used with Envision + FLEX Mouse [LINKER] (K8021, Dako). Rabbit polyclonal IgG (cat. no. ab27478, Abcam, Cambridge, UK) was used as isotype control for CCL20 and CCR6, while Mouse monoclonal IgG1 and IgG2a (cat. no. X0931 and X0943, Dako, Glostrup, Denmark)) were used as isotype controls for CD45RO and CD1α, and CD3 and CD20, respectively. Isotype controls were performed together with negative control (primary antibody omitted). Secondary antibody was from Dako Real Envision (rabbit/mouse), and the Dako EnVision peroxidase kit and Dako DAB+ chromogen (Dako, Glostrup, Denmark) were used for detection together with hematoxylin counterstaining. *In situ* hybridization (ISH) for *CCL20* and *CCR6* mRNA was done on the same colonic biopsies as the immunohistochemical staining, using custom probes for *CCL20* and *CCR6*, UbC as positive control and DapB as negative control together with the RNAScope 2.0 kit (all from Advanced Cell Diagnostics, Hayward, CA, USA). Procedures were according to the manufacturer’s protocol, and sections were counterstained with hematoxylin.

The absolute and relative numbers of CCR6 staining cells were assessed by counting CCR6 positive and total number of lamina propria cells. In each section ten different areas of lamina propria measuring at least 15,000 μm^2^ each were counted. All areas were selected to secure counts over the whole depth of lamina propria, i.e. from the basement membrane of the surface epithelium to the muscularis mucosae. This was performed for both CCR6 mRNA and protein, and fraction of *CCR6* positive epithelial cells overlying the lamina propria areas was also determined. As a measure of the amount of *CCR6* mRNA that is translated into protein CCR6, we used the ratio between IHC CCR6 positive cells and ISH *CCR6* positive cells. Colocalization of mRNA and protein was assessed for CCR6 by performing ISH and IHC on serial (neighbouring) sections. Serial sections were also used to analyse colocalization of CCR6 or CCL20 positive cells in lamina propria with immune cell markers.

#### 2.3.4 Stimulation assay in the HT29 cell line

The human epithelial cell line HT29 (colorectal carcinoma, Cat. No. HTB-38, lot no. 59561256) (ATCC, Manassas, VA, USA) was used to investigate mechanisms for expression and release of CCL20. The cells were cultured in RPMI with 10% fetal bovine serum, glutamine 2mM and gentamicin 0.05%, at 37°C and 5% CO2. All functional cell assays were done in the same medium, without gentamicin. Cells were detached from the cell culture flask using trypsin/EDTA, resuspended in culture medium and counted using a Countess Automated Cell Counter (Life Technologies, Grand Island, NY, USA). Flat-bottom 96-well plates seeded with 20,000 cells were used for the experiments, which were done in triplicate. The plates were incubated overnight at 37°C with 5% CO2, before medium was removed and fresh medium applied with or without PRR ligand/cytokine. The cells were stimulated for 20 hours, and the supernatant for all experiments harvested and stored at -20°C until analysis.

The following ligands and concentrations were used for the cell experiments: Lipopeptide Pam3CysSK4 (P3C) (TLR2/1) 300 ng/mL, lipomannan (LM) (TLR2/6) 30 ng/mL, lipopolysaccharide (LPS) (TLR4) 100 ng/mL Flagellin (TLR5) 100 ng/mL, the antiviral compound R848 (TLR7/8) 100ng/mL, the peptidoglycan component muramyl dipeptide (MDP) (NOD2) 1μg/mL (all from InvivoGen, Toulouse, France); synthetic double-stranded RNA mimic polyinosinic:polycytidylic acid (poly (I:C)) (TLR3) 0.5, 5 or 50 μg/mL (Amersham Bioscience, Piscataway, NJ, USA), unmethylated CpG dinucleotides (TLR9) 10 μM (TibMolBiol, Berlin, Germany), and the recombinant human cytokines IL-10 100 ng/mL and IL-1β 100 ng/mL (both from PeproTech, Rocky Hill, NJ, USA).

#### 2.3.5 TLR3 receptor silencing

For transfection experiments, 20,000 HT29 cells were grown in 96 well flat bottom platens (in medium without gentamicin). Transfection was done using Lipofectamin, RNAiMAX (Ambion, Invitrogen Dynal, Waltham, MA, USA) and *TLR3* small interfering RNA (siRNA), siTLR3.6 and siTLR3.8 or a nonsilencing control RNA (nsRNA) (all 5nM) (all from Qiagen, Venlo, Netherlands). The cells were transfected for 24 hours at 37°C with 5% CO2, followed by a 12 hour stimulation with 5μg/mL poly (I:C) before collecting the supernatant. The cells were either assayed for viability using the MTT assay, or lysed and kept at -80°C for isolation of RNA and qRT-PCR to confirm siRNA knockout of TLR3.

The Ambion mirVana miRNA kit was used to isolate total RNA from the HT29 cell lysates, and cDNA generated using the High capacity RNA-to-cDNA kit before qRT-PCR with the TaqMan gene expression assay for *TLR3* (probe ID Hs01551078_m1), *CCL20* (probe ID Hs01011368_m1) and *GAPDH* (probe ID Hs99999905_m1). The qRT-PCR was done on a StepOnePlus Real-Time PCR System with StepOne software v. 2.1. *TLR3* and *CCL20* mRNA abundance were normalized to the housekeeping gene *GAPDH*, and expression of both measured relative to the negative control, which were HT29 cells transfected with nsRNA.

To assess the viability of the transfected cells 0.5mg/mL MTT [3-(4,5-dimethylthiazol-2-yl)-2,5-diphenyl tetrazolium bromide] (Promega, Nacka, Sweden) in 10% FCS/RPMI was added to the wells, before incubation at 37°C in 5% CO2 for 3 hours. The supernatant was then replaced with alkaline isopropanol with 0.25% 1M NHOH and optical density measured at 570 nm after 30 min stirring using the luminometer function of the Walla Victor 3 1420 Multilabel Counter from Perkin Elmer (Boston, MA, USA).

Supernatants from every HT29 assays were analysed for CCL20 using a commercially available enzyme-linked immunosorbent assay (ELISA) human Duo-Set (R&D, Abingdon, UK), according to the manufacturer’s instructions.

### 2.4 Statistical analysis

Microarray data analysis was done with Bioconductor [25] in the R software environment. Linear models with least squares regression and empirical Bayes moderated t-statistics was used to determine the differential expression [26]. To adjust the p-values for multiple comparisons, Benjamini Hochberg false discovery rate correction was performed. Data from counting of cells were normalized, and analysed by ANOVA with multiple comparisons correction (Holm-Sidak). The data from analysing supernatant from stimulation assays were analysed using Kruskal-Wallis nonparametric test with Dunn’s multiple comparisons post-test. Mann-Whitney U-test was used to detect group differences, while rRT-PCR results (calculated by the ΔΔCT method) from transfection assays and colonic biopsies were analysed by paired and unpaired t-test, respectively. Statistics were performed in GraphPad Prism 6.0 (GraphPad Software Inc., San Diego, CA, USA).

## Results

### 3.1 CCL20 and CCR6 mRNAs are overexpressed in active IBD

**Fig 1:**
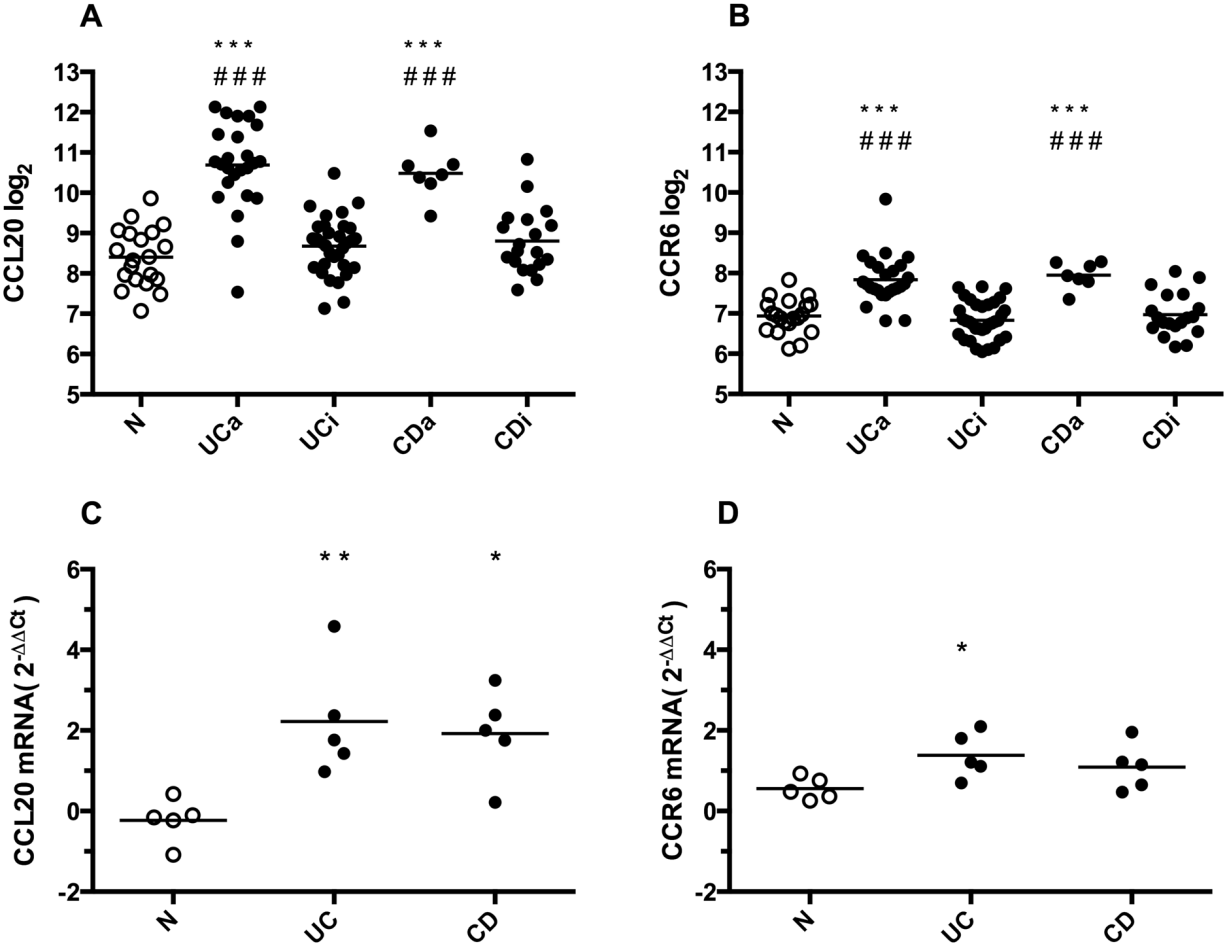
*CCL20* and *CCR6* gene expression in colonic biopsies. **A and B:** Microarray gene expression results of *CCL20* and *CCR6* in colonic biopsies from healthy controls (N), active (UCa) or inactive (UCi) ulcerative colitis, and active (CDa) or inactive (CDi) Crohn’s disease. Individual values (Log_2_) and mean are plotted. **C and D:** qRT-PCR gene expression results of *CCL20* and *CCR6* in colonic biopsies from N, Ulcerative Colitis (UC) and Crohn’s disease (CD), n = 5 in each group. Individual values (foldchange 2^-ΔΔCt^) and mean are plotted. *p<0.05 versus N, **p<0.01 versus N, ***p<0.001 versus N, ###p<0.001 versus inactive disease.

The patient characteristics and microarray results have been reported previously [27, 28]. Fig 1 shows the microarray and qRT-PCR gene expression levels for *CCL20* and *CCR6*. Compared to N, *CCL20* is clearly overexpressed in both UCa (log2 ratio 2.43 with fold change 5.39, p<0.001) and CDa (log2 ratio 2.08 with fold change 4.23, p<0.001). The same is seen for *CCR6* in UCa (log2 ratio 0.87 with fold change 1.83, p<0.001) and CDa (log2 ratio 1.01 with fold change 2.01, p<0.001). In colonic biopsies from areas with no sign of active disease (UCi and CDi), there was no significant difference in expression levels compared to N.

Five biopsies from each group were randomly selected for qRT-PCR verification of the microarray results. These analyses confirmed the microarray data for *CCL20*, with significant differences UCa vs N and CDa vs N (both p<0.01). For *CCR6*, qRT-PCR confirmed the UCa vs N (p<0.05), while the CDa vs N comparison had a marginal p value of 0.06.

### 3.2 CCL20 and CCR6 mRNA and protein is localised to epithelium and infiltrating cells

**Fig 2:**
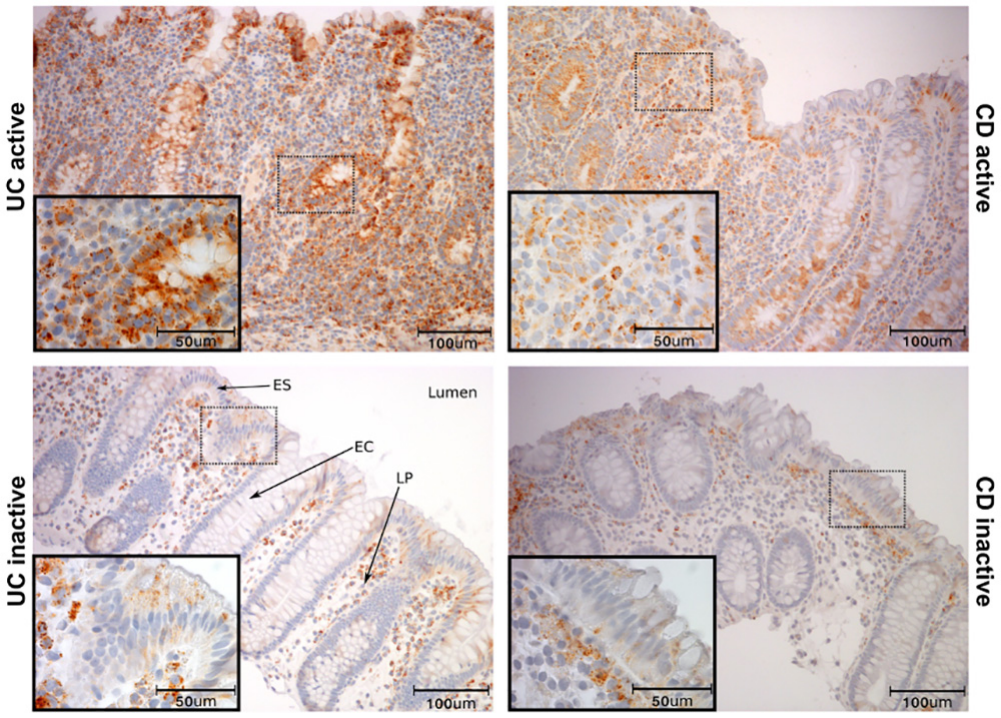
Localization of CCL20 protein in colonic biopsies. Immunohistochemical staining for CCL20 protein in colonic biopsies from active (UCa) or inactive (UCi) ulcerative colitis, and active (CDa) or inactive (CDi) Crohn’s disease. Healthy controls display CCL20 positivity similar to uninflamed biopsies and are not shown. Scale bars as indicated. ES = Surface epithelium, EC = Crypt epithelium, LP = Lamina propria.

**Fig 3:**
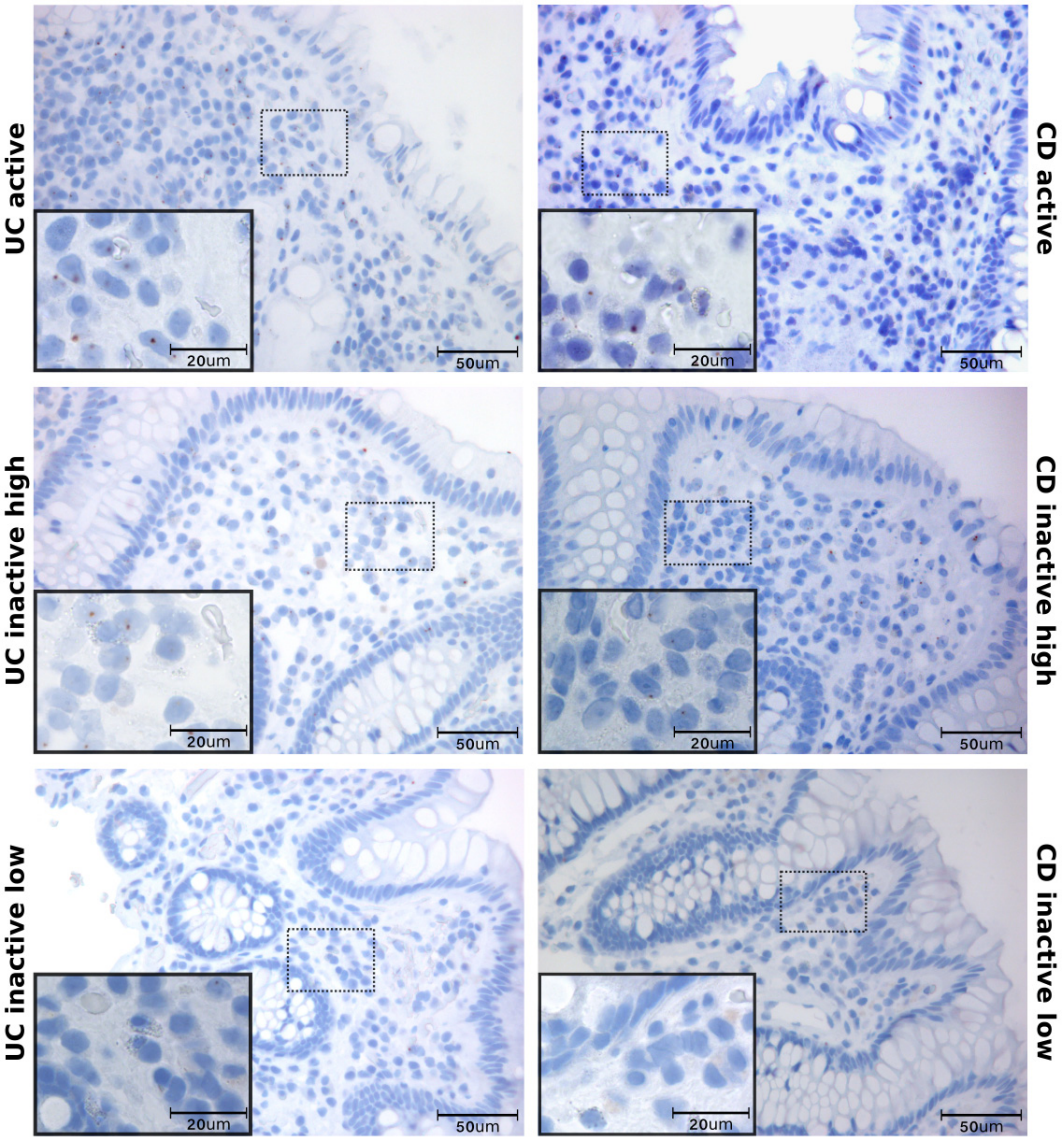
Localization of *CCR6* mRNA in colonic biopsies. In situ hybridization of *CCR6* mRNA in colonic inflammatory bowel disease tissue. Sections are taken from biopsies active (UCa) or inactive (UCi) ulcerative colitis, and active (CDa) or inactive (CDi) Crohn’s disease. High and low expression of *CCR6* in inactive disease is shown. Scale bars as indicated.

**Fig 4:**
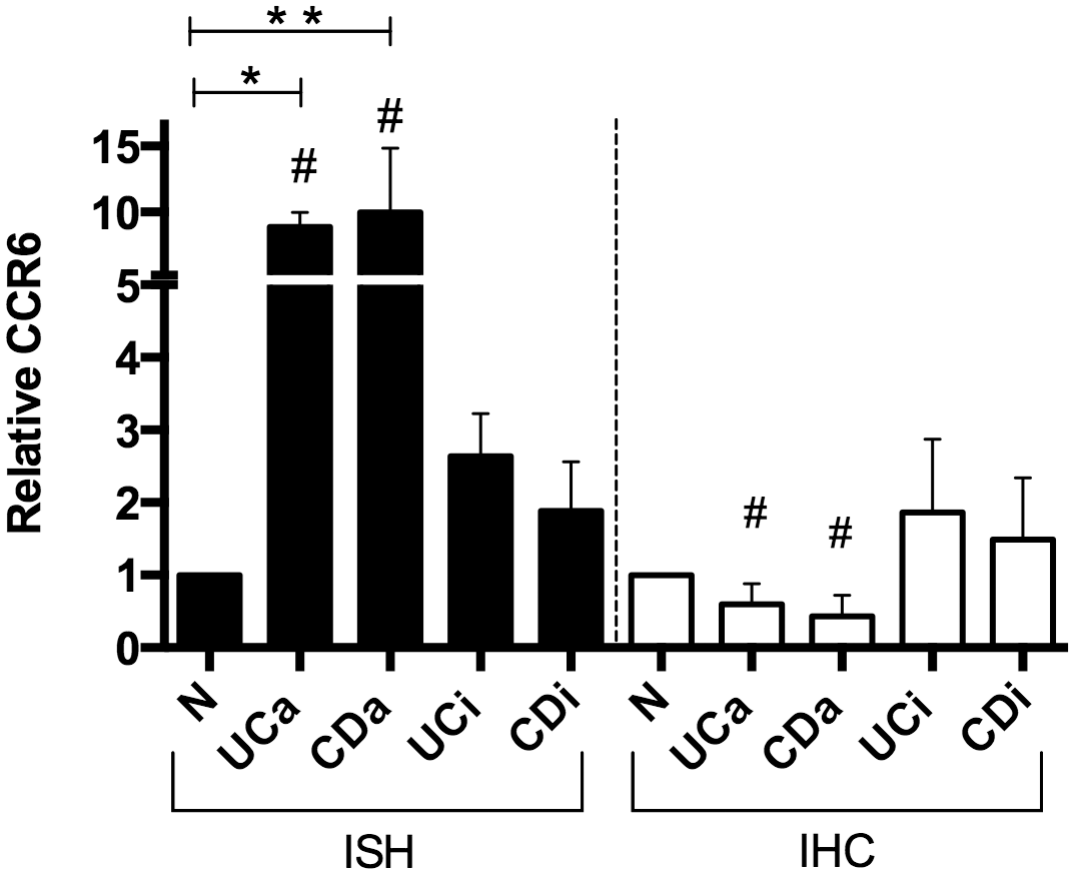
Number of CCR6 mRNA and protein positive cells in lamina propria. Number of CCR6 immunohistochemically (IHC) and *in situ* hybridization (ISH) positive cells in colonic biopsies from healthy controls (N), active (UCa) or inactive (UCi) ulcerative colitis, and active (CDa) or inactive (CDi) Crohn’s disease. Five subjects in each group for IHC, and three for ISH. CCR6 positive cells were counted in ten 15,000μm^2^ areas of lamina propria for each section. * p< 0.05, ** p<0.01, # p<0.05 versus corresponding inactive disease group. Mean ± SD shown.

**Fig 5:**
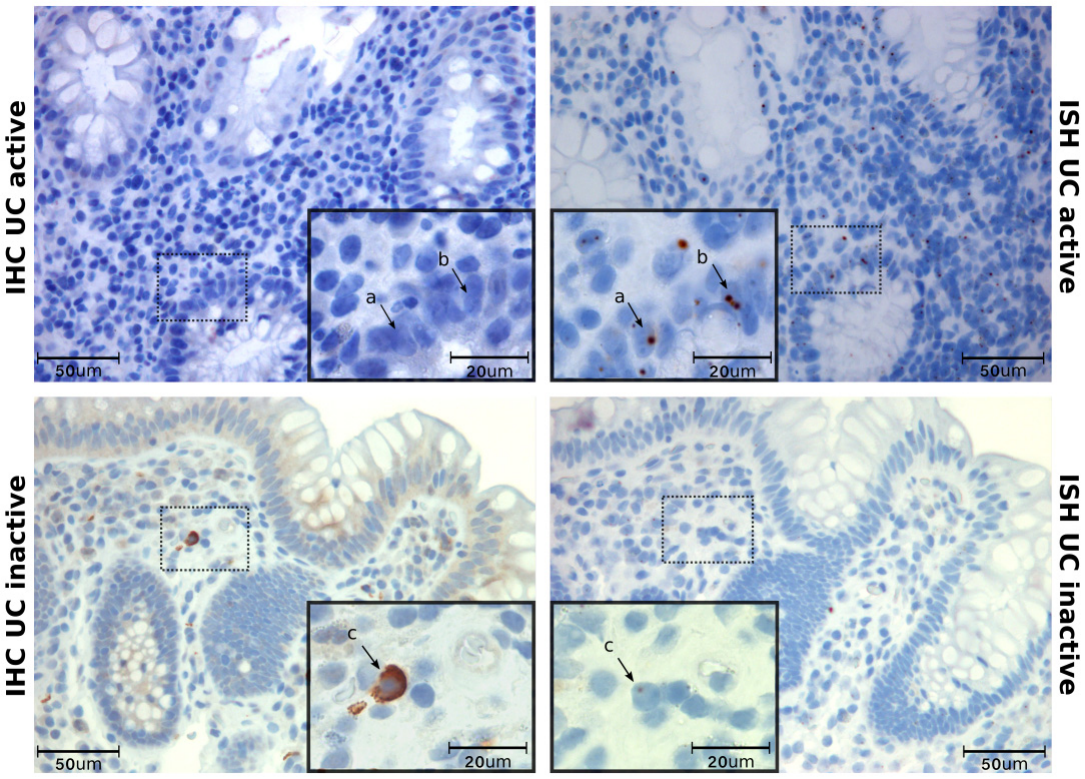
CCR6 protein and mRNA in serial sections. Immunohistochemistry (IHC) and in situ hybridization (ISH) show CCR6 protein and mRNA in colonic biopsies from active UC and inactive CD. Serial sections from the same biopsy were used to compare the localization of mRNA and protein. Arrows show that there is no overlap of protein and mRNA in active disease (a, b), while a clear overlap is seen in inactive disease(c). Scale bars as indicated.

**Fig 6:**
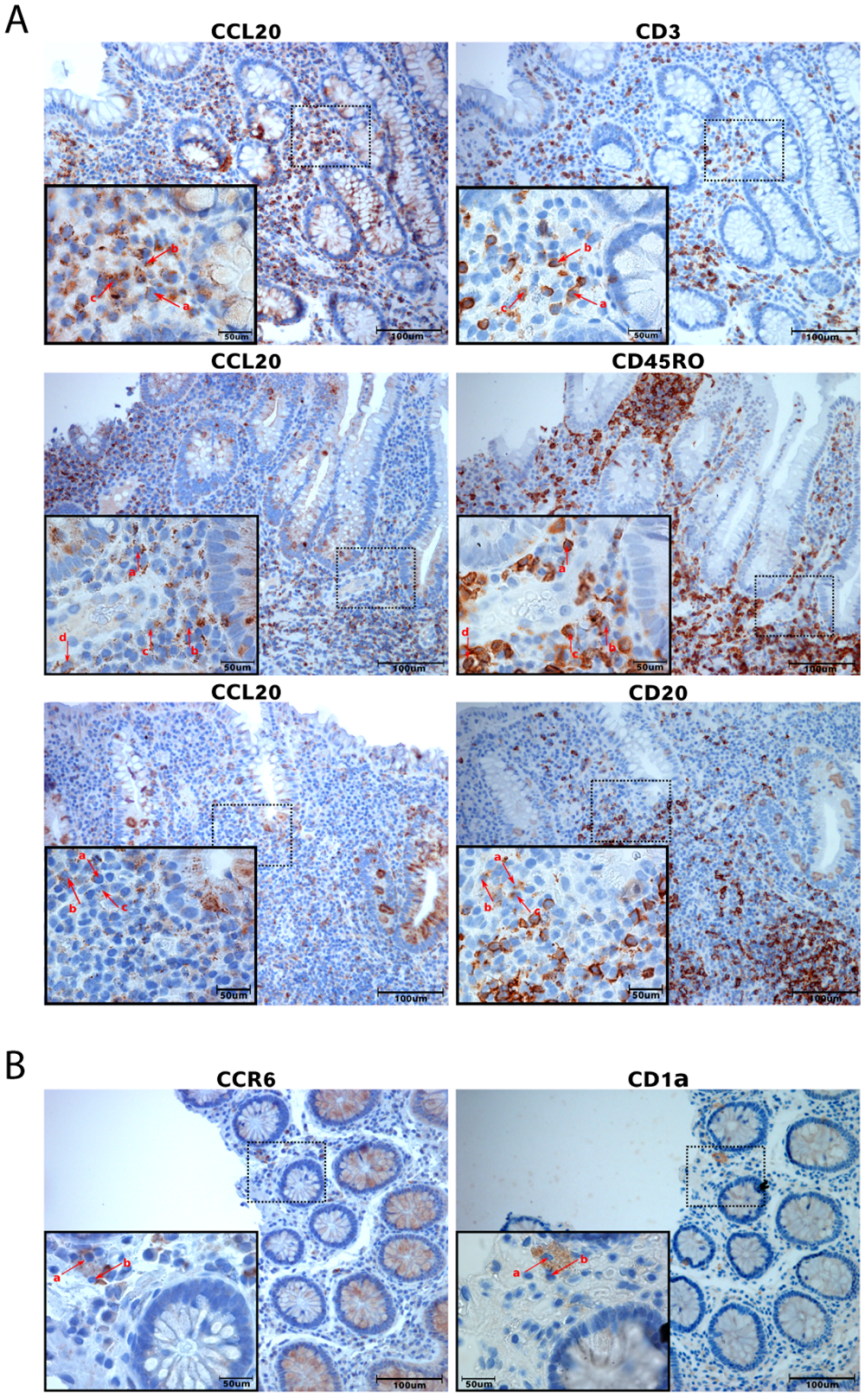
Immune cell typing of CCL20 and CCR6 positive cells in serial sections. Immunohistochemistry on serial sections from the same biopsy was performed to localize CCR6 and CCL20 positivity to different immune cells. **A:** Sections from biopsies from active disease were used for CCL20 investigations. There is a partial overlap between CCL20+ cells and CD3+, CD45RO+ and CD20+ cells (arrows a, b, c, d). **B:** Sections from biopsies from inactive disease for CCR6. There is a partial overlap between CCR6+ cells and CD1α+ cells (arrows a, b). Scale bars as indicated.

Five randomly chosen colonic biopsies from each group were used for IHC detection of CCL20 (Fig 2). Staining was specific and with negative isotype controls. A very weak CCL20 staining was seen in the surface epithelial layer in inactive disease and in healthy individuals. In UCa and CDa, on the other hand, epithelial CCL20 positivity was strong and seen throughout the epithelium including crypts. Scattered CCL20 positive mononuclear cells, with lymphocyte and plasma cell morphology, were also seen in the lamina propria. While these inflammatory cells form a subepithelial band in inactive UC and CD, a massive infiltration of CCL20 positive immune cells can be found throughout the lamina propria in active UC and CD. ISH showed a distribution of *CCL20* mRNA similar to IHC findings in all groups (S1 Fig), but also showed that mRNA localized to follicle-associated epithelium (FAE) in healthy controls (S2 Fig). Within the groups of inactive IBD, *CCR6* mRNA displayed a considerable sample-to-sample variation (Fig 3). Control biopsies from healthy individuals showed little to no *CCR6* expression (S3 Fig). IHC staining showed cells positive for CCR6 protein in the lamina propria, morphologically resembling lymphocytes, plasma cells, and dendritic cells. The epithelial layer had scattered weakly positive cells, which could be unspecific since isotype and negative controls both showed a few weakly positive epithelial cells. However, ISH positive cells were also found in the epithelium, and the negative controls showed no staining. The amount of cells positive for *CCR6* mRNA in the lamina propria were significantly higher in active disease groups compared to inactive and control groups, while the amount of cells positive for CCR6 protein was significantly lower in active disease groups compared to inactive disease groups but not healthy controls (Table 2, Fig 4). In the epithelium, the fraction of *CCR6* positive cells was largest in active disease groups, and this reached statistical significance for active CD compared to healthy controls (N: 1.49±0.43%, aUC: 9.90±7.75%, aCD: 11.93±0.33%, iUC: 4.30±0.93%, iCD: 3.36±0.88%. p<0.05 aCD vs. N). To investigate this further, we performed ISH and IHC for CCR6 mRNA and protein on serial sections (Fig 5). This showed that there is an overlap between cells positive for CCR6 mRNA and protein in inactive disease and healthy controls, while this is overlap is much less consistent in active disease. These results were supported by comparing the number of IHC and ISH CCR6 positive cells in all disease groups, which showed that the relationship between CCR6 mRNA and protein positivity is stronger in inactive than in active disease (Table 2). In the colocalization assays for CCL20 and CCR6 protein with immune cell markers, we found that CCL20 positive cells overlap with immune cell markers for T-cells (CD3), activated T-cells (CD45RO) and B-cells (CD20) (Fig 6A). For CCR6 positive cells, we found overlap with markers for immature dendritic cells (CD1α) (Fig 6B).

**Table 2:** Counting of CCR6 positive cells in Immunohistochemistry and *in situ* hybridization.

	IHC pos. cells	ISH pos. cells	IHC/ISH
N	4.9±1.1	4.3±2.0	1.14±0.27
UCa	2.7±0.94 #	38.2±20.9 * #	0.071±0.025 ** ##
CDa	1.9±1.1 #	36.6±3.0 ** #	0.052±0.030 ** ##
UCi	8.7±4.3	10.8±3.8	0.81±0.40
CDi	6.7±3.0	7.2±1.6	0.93±0.41

Number of positive staining cells given in mean ± SD. N = controls, UC = Ulcerative Colitis, CD = Crohn's Disease, a = active, i = inactive.

* p<0.05 versus control.

** p<0.01 versus control.

^#^ p<0.05 versus inactive disease.

^##^ p<0.01 versus inactive disease.

### 3.3 TLR3 stimulation potently induces CCL20 release from HT29 cells

**Fig 7:**
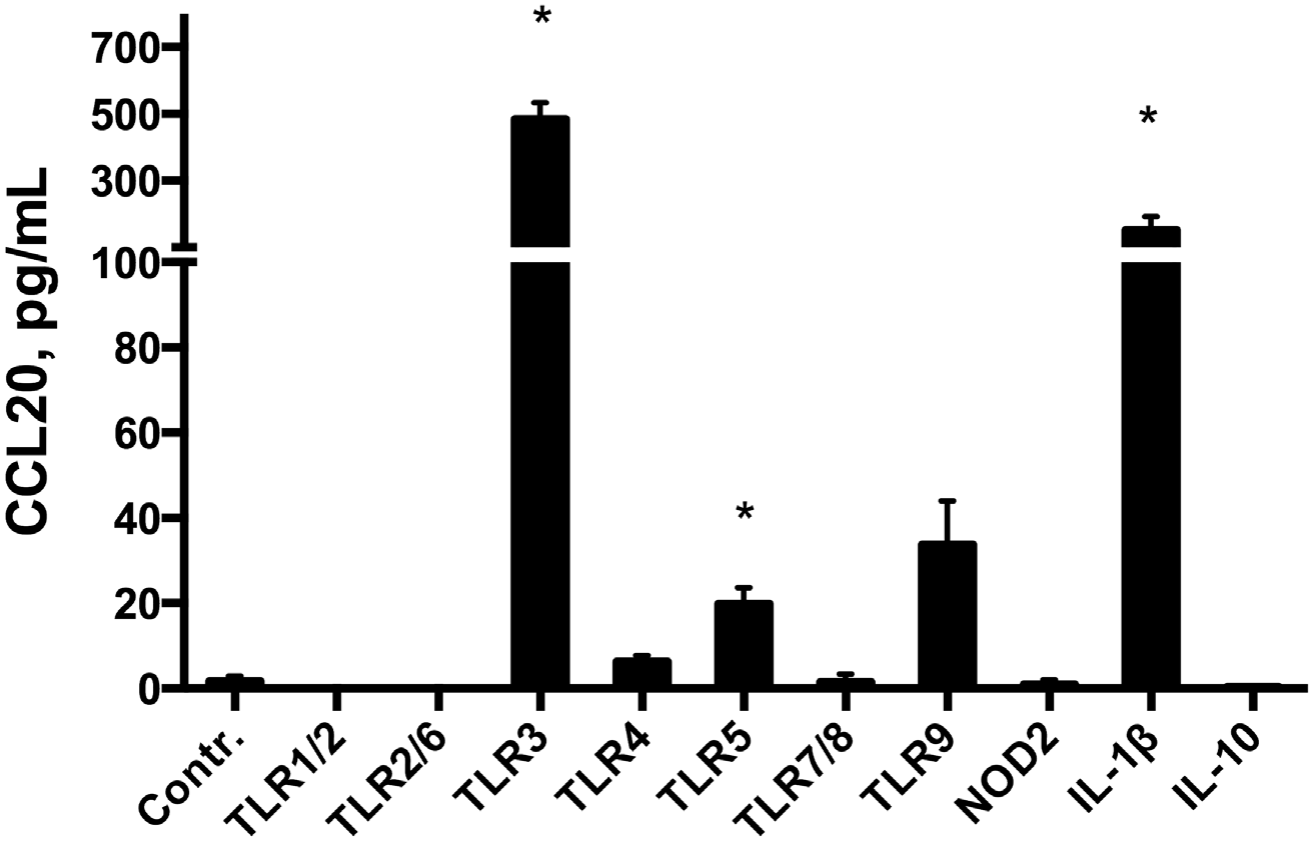
CCL20 release from HT29 cells in PRR stimulation assay. Stimulation assay in the HT29 cell line. CCL20 release after 20 hour stimulation with ligands for pattern recognition receptors (PRRs) from left to right, TLR-1–9 and NOD2; and IL-10 and IL-1β for 20 hours. Control is medium alone. Abbreviations, PRR ligands and concentrations used are given in material and methods. * p<0.05 versus medium. Mean ± SD is shown.

The predominantly epithelial localization of CCL20 and induction during active inflammation prompted us to examine possible mechanisms for this epithelial response using a panel of PRR ligands and proinflammatory signal substances. The TLR5 ligand Flagellin, the TLR3 ligand poly (I:C) and the proinflammatory cytokine IL-1β (Fig 7) all induced a significant release of CCL20. The poly (I:C) response was substantial with a 446-fold increase in CCL20 release, while the CCL20 was 9-fold increased after Flagellin stimulus. IL-1β increased CCL20 release 140-fold.

**Fig 8:**
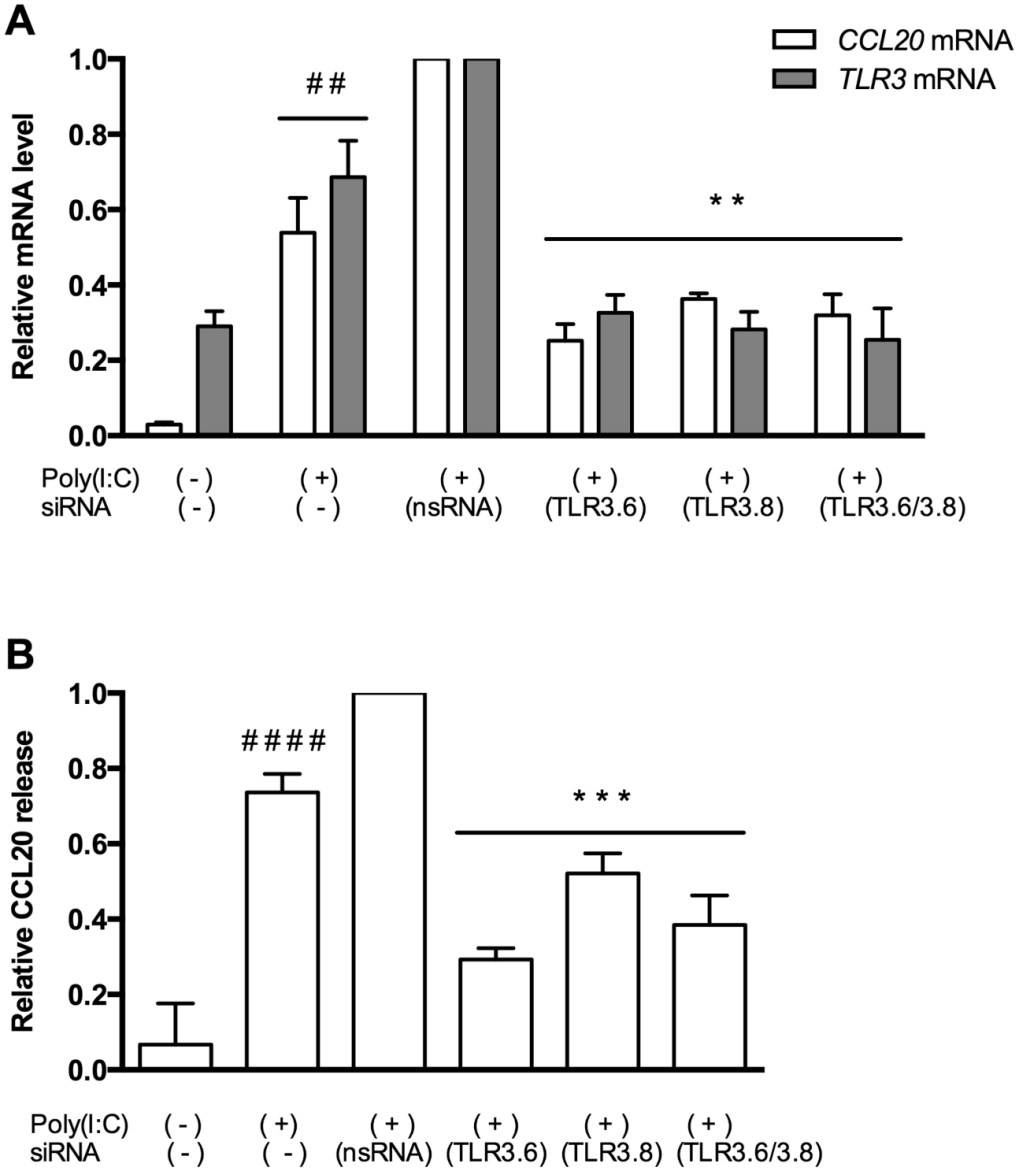
CCL20 gene expression and protein release in TLR3 transfection assay. **A:** *TLR3* and *CCL20* mRNA abundance in poly (I:C) stimulated HT29 cells with and without TLR3 small interfering RNA(siRNA) transfection. Non-signalling siRNA is designated nsRNA, two different TLR3 specific siRNAs (TLR3.6 and TLR3.8) were used alone or in combination. The cells were transfected for 24 hours using TLR3 siRNAs or nsRNA and then stimulated with the TLR3 ligand poly (I:C) for 20 hours. Controls were unstimulated untranfected cells, poly (I:C) stimulated cells and cells treated with nsRNA stimulated with poly (I:C). ** p< 0.01 versus nsRNA, ## p<0.01 versus unstimulated control. Mean ± SD of triplicated is shown. **B:** The poly (I:C) effect on CCL20 release in HT29 cells after transfection with TLR3 siRNAs as described above. *** p< 0.001 versus nsRNA, #### p<0.0001 versus unstimulated control. Mean ± SD of triplicates is shown.

Since the cell stimulation experiments showed a potent effect of the TLR3 ligand poly (I:C), we performed *TLR3* siRNA experiments to further investigate the role of TLR3 in the CCL20 response (Fig 8). Transfection did not completely ablate *TLR3* expression; *TLR3* abundance was reduced by 67.4%, 71.8% and 74.6% after treatment with the blocking agents siTLR3.6, siTLR3.8 and the combination of the two. Expression of *CCL20* was reduced by 74.8%, 63.7% and 68.0%, and correspondingly CCL20 release from the cells was reduced by 70.7%, 47.9% and 61.6%, but neither to the level of unstimulated controls.

The assay also showed that through poly (I:C) stimulation of the HT29 cell, *TLR3* expression increased by 240%, *CCL20* expression by 1798% and CCL20 release increased by 1098%. The MTT assay showed unchanged viability for all interventions as compared to control cells (results not shown). These results demonstrate that the CCL20 response to poly (I:C) in HT-29 cells is indeed mediated through a TLR3 pathway.

## Discussion

The role of CCL20 in IBD has been investigated previously, using diverse methods and clinical materials of variable size [29–32]. Existing knowledge is somewhat conflicting, both with regard to whether only ulcerative colitis or Crohn’s disease, or both forms of IBD, exhibit CCL20 involvement, and whether this is found only in active disease or not [30–32]. Data on the regulation of CCL20 expression in the intestinal mucosa and the involvement of CCR6 is also scarce [32]. With the CCL20-CCR6 axis emerging as a possible drug target, further studies of these aspects of IBD pathobiology are indeed warranted [10–12].

The basis for the studies presented here is a genome-wide transcriptome analysis of a very well controlled material of colonic mucosal samples from active and inactive ulcerative colitis and Crohn’s disease, and healthy controls. This material is one of the largest existing, and showed excellent performance in a meta-analysis of comparable datasets [3]. The transcriptome analysis shows that expression of *CCL20* is potently induced in active ulcerative colitis and active Crohn’s disease, while expression levels in inactive disease are indistinguishable from healthy controls. This is somewhat at odds with previous results, which have shown either *CCL20* overexpression in only Crohn’s disease and not ulcerative colitis [30], or also in inactive disease [31]. We think, however, that the results presented here are very robust due to the size and meticulous control of the clinical material. Moreover, our results are based on two modes of mRNA quantitation (gene expression microarrays and qRT-PCR), as well as ISH, rather than only qRT-PCR as in the previous publications. In the presented material *CCR6* is also significantly higher expressed in active ulcerative colitis and active Crohn’s disease as compared with inactive disease or healthy controls, with no significant difference in expression level between inactive disease and healthy individuals. In our opinion, these parallel changes in ligand and receptor mRNA abundances further underline the robustness of the transcriptome data.

We further examined the localization of CCL20 and CCR6 mRNA and protein in a randomly chosen subset of the mucosal samples used for gene expression studies, and we found that CCL20 mRNA and protein were massively upregulated in the epithelial cells during active inflammation. *CCL20* expression could also be seen in infiltrating cells of the lamina propria. In healthy controls *CCL20* could be mapped mostly to follicle-associated epithelium, which is in accordance with the findings by Kaser et al. [31]. The clear upregulation of CCL20 protein and mRNA seen in the epithelial cells in the present study strongly indicates that epithelial mechanisms are central in the CCL20 involvement in active IBD.

*CCR6* mRNA was localized to infiltrating cells and epithelium, and clearly more abundant in active UC and CD as compared to inactive disease and controls. Unlike CCL20, the CCR6 IHC staining pattern was strikingly different from ISH, showing a lower number of IHC positive cells in active IBD than in inactive disease and normal controls. The ISH results corresponded well with *CCR6* gene expression measured both by microarray and qRT-PCR. The difference between mRNA and protein expression patterns may be a consequence of ligand-receptor complex internalization, which is a phenomenon known to occur when CCL20 binds CCR6 [33, 34]. With the large amounts of CCL20 available in the active disease groups, it is not unreasonable to believe that internalization leads to a false impression of fewer CCR6 positive cells in IHC on these samples. Another possible explanation is that saturation of receptors renders the receptor protein inaccessible for the CCR6 antibody. Previous findings suggest that CCR6 is expressed only in the epithelium of the intestinal mucosa [35], or only in infiltrating immune cells [16]. Internalisation could explain the discrepancies in earlier studies, and their inconsistence with our findings. Because the number of CCR6 positive cells in IHC was highest in inactive disease, we chose to study overlap with immune cell markers here rather than in active disease. For CCL20, the opposite was true, and colocalization assays were performed on sections from active disease biopsies. Our results show that in lamina propria, lymphocytes are the cells that mainly colocalize with CCL20 while immature dendritic cells colocalize with CCR6 protein. However, none of the overlaps between immune cell markers and CCL20 or CCR6 were complete, and what additional cell types produce CCR6 and CCL20 protein still needs further investigation. IHC cannot prove whether a protein is produced in a cell or internalized through receptor binding, but our studies show that the CCL20 positivity that can be found in the lamina propria of IBD patients is at least related to lymphocytes, and the CCR6 positivity to immature dendritic cells.

The importance of the innate immune system in the pathogenesis of IBD and how TLRs are involved in shaping mucosal immune system is becoming more evident [6, 36]. To our knowledge, involvement of the innate immune system in the regulation of human CCL20 expression has previously not been examined thoroughly. Earlier studies have used the signalling molecules IL-1α, IL-1β and TNF-α and found that these stimulate synthesis and release of CCL20 from the epithelium and from peripheral blood mononuclear cells [16, 29, 35, 37]. Sugiura et al. found that TLR1 stimulation by Yersinia Enterocolitica induce CCL20 release in intestinal mucosa of mice and in Caco2 cells transfected with TLR1 [23]. We saw no effect of TLR1 stimulation of HT29 cells. The results from our HT29 cell studies show that two members of the TLR system stimulate CCL20 release, flagellin (TLR5) and poly (I:C) (TLR3). In particular, the TLR3 response was very potent. Underlining this, in a series of separate studies on HT29 cells we found that CCL20 is the fifth most potent response to poly (I:C) stimulation, mRNA abundance being 48-fold increased (unpublished data). TLR3 stimulation in combination with IL-1β has previously been shown to initiate CCL20 upregulation in human gingival fibroblasts in periodontal disease [24], and our results indicate that this cellular mechanism can also be important in IBD. Our novel observations, together with previous results from our laboratory showing that TLR3 is upregulated in inflammatory bowel disease [38], strongly indicate that this PRR may indeed play a role in activating the CCL20-CCR6 axis in inflammatory bowel disease. The ligands for TLR3 signalling in IBD are at present unknown. TLR3 senses dsRNA, which is found during replication of most viruses, in addition to the dsRNA viruses themselves. However, there is also evidence that TLR3 senses endogenous mRNA from damaged tissue and might maintain inflammation independently of viral infection [39, 40]. Blocking *TLR3* mRNA did not completely obliterate CCL20 expression or release from the HT29 cells, which underlines that there are several mechanisms for CCL20 production in intestinal epithelial cells. Moreover, residual *TLR3* expression, as siRNA assays did not block *TLR3* expression entirely, may also contribute to this. We therefore conclude that the TLR3 pathway certainly is involved.

In conclusion, this study shows that epithelial CCL20 is potently upregulated in active ulcerative colitis and Crohn’s disease, but not in inactive disease. A CCL20-CCR6 axis indeed seems to be present, and the innate immune system is involved in CCL20 induction in the epithelium particularly through TLR3. Human *ex vivo* and animal *in vivo* models are needed to further investigate the mechanism of TLR3 induced CCL20 production in the intestinal epithelium.

## Supporting Information

S1 Fig Localization of *CCL20* mRNA in colonic biopsies. *In situ* hybridization for localization of *CCL20* mRNA in colonic biopsies from active (UCa), inactive (UCi) ulcerative colitis and healthy controls. Scale bars as indicated.
(TIF)

S2 Fig *CCL20* mRNA in follicle associated epithelium (FAE). *In situ* hybridization staining for *CCL20* mRNA in colonic tissue from a healthy control, including a lymphoid follicle and follicle-associated epithelium (FAE) staining positive for *CCL20* mRNA. Scale bars as indicated.
(TIF)

S3 Fig *CCR6* mRNA in colonic biopsy from healthy control. *In situ* hybridization staining for *CCR6* mRNA in colonic tissue from a healthy control, showing staining pattern similar to inactive disease groups with low positivity for *CCR6* mRNA (Fig 3). Scale bars as indicated.
(TIF)
