# Supplementary figures and images for: Expression of CCL20 and Its Corresponding Receptor CCR6 Is Enhanced in Active Inflammatory Bowel Disease, and TLR3 Mediates CCL20 Expression in Colonic Epithelial Cells
Source: PLoS One. 2015 Nov 4;10(11):e0141710. doi: 10.1371/journal.pone.0141710 (PMC4633243; doi:10.1371/journal.pone.0141710)

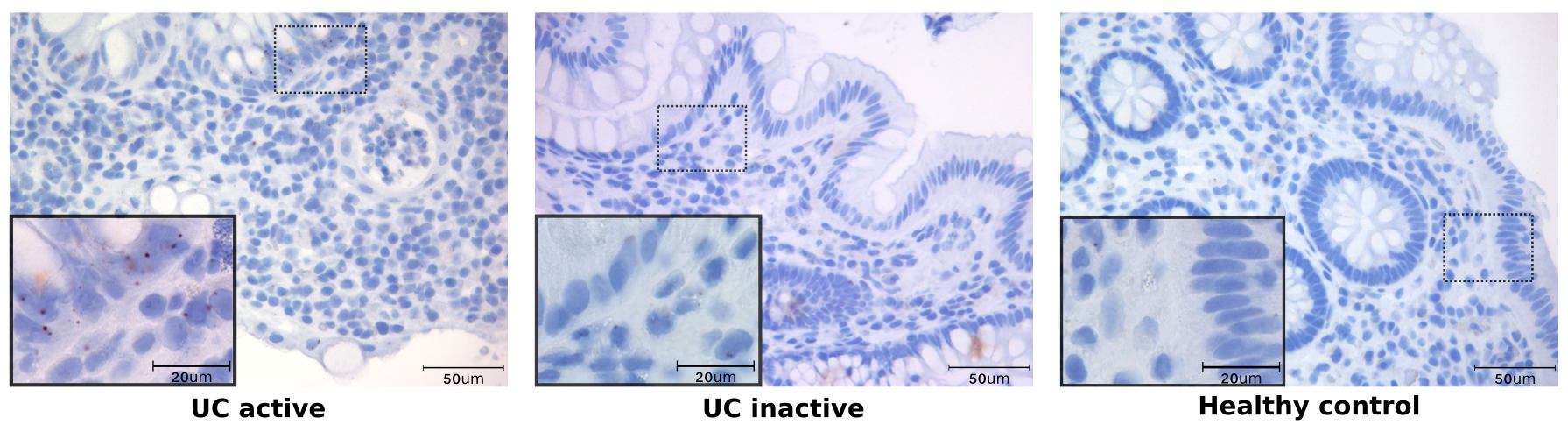

Supplement: S1 Fig — In situ hybridization for localization of CCL20 mRNA in colonic biopsies from active (UCa), inactive (UCi) ulcerative colitis and healthy controls. Scale bars as indicated. (TIF) [file pone.0141710.s001.tif]

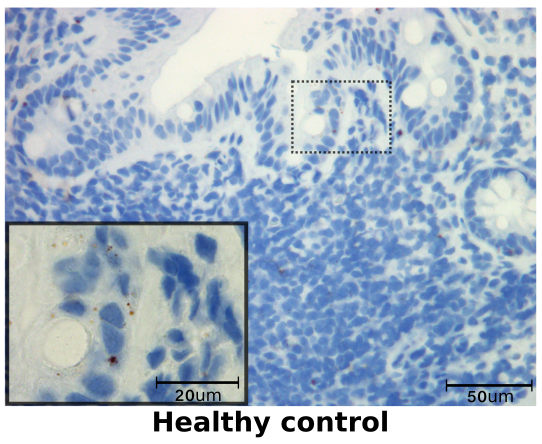

Supplement: S2 Fig — In situ hybridization staining for CCL20 mRNA in colonic tissue from a healthy control, including a lymphoid follicle and follicle-associated epithelium (FAE) staining positive for CCL20 mRNA. Scale bars as indicated. (TIF) [file pone.0141710.s002.tif]

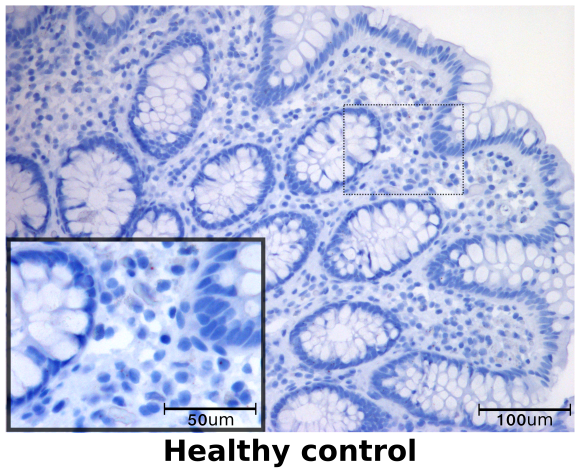

Supplement: S3 Fig — In situ hybridization staining for CCR6 mRNA in colonic tissue from a healthy control, showing staining pattern similar to inactive disease groups with low positivity for CCR6 mRNA (Fig 3). Scale bars as indicated. (TIF) [file pone.0141710.s003.tif]
